# Differentiation of Low-Grade Astrocytoma From Anaplastic Astrocytoma Using Radiomics-Based Machine Learning Techniques

**DOI:** 10.3389/fonc.2021.521313

**Published:** 2021-06-01

**Authors:** Boran Chen, Chaoyue Chen, Jian Wang, Yuen Teng, Xuelei Ma, Jianguo Xu

**Affiliations:** ^1^ Department of Neurosurgery, West China Hospital, Sichuan University, Chengdu, China; ^2^ State Key Laboratory of Biotherapy and Cancer Center, West China Hospital, Sichuan University, and Collaborative Innovation Center for Biotherapy, Chengdu, China; ^3^ School of Computer Science, Nanjing University of Science and Technology, Nanjing, China; ^4^ Department of Biotherapy, Cancer Center, West China Hospital, Sichuan University, Chengdu, China

**Keywords:** machine learning, glioma, astrocytoma, texture analysis, radiomics

## Abstract

**Purpose:**

To investigate the diagnostic ability of radiomics-based machine learning in differentiating atypical low-grade astrocytoma (LGA) from anaplastic astrocytoma (AA).

**Methods:**

The current study involved 175 patients diagnosed with LGA (n = 95) or AA (n = 80) and treated in the Neurosurgery Department of West China Hospital from April 2010 to December 2019. Radiomics features were extracted from pre-treatment contrast-enhanced T1 weighted imaging (T1C). Nine diagnostic models were established with three selection methods [Distance Correlation, least absolute shrinkage, and selection operator (LASSO), and Gradient Boosting Decision Tree (GBDT)] and three classification algorithms [Linear Discriminant Analysis (LDA), Support Vector Machine (SVM), and random forest (RF)]. The sensitivity, specificity, accuracy, and areas under receiver operating characteristic curve (AUC) of each model were calculated. Diagnostic ability of each model was evaluated based on these indexes.

**Results:**

Nine radiomics-based machine learning models with promising diagnostic performances were established. For LDA-based models, the optimal one was the combination of LASSO + LDA with AUC of 0.825. For SVM-based modes, Distance Correlation + SVM represented the most promising diagnostic performance with AUC of 0.808. And for RF-based models, Distance Correlation + RF were observed to be the optimal model with AUC of 0.821.

**Conclusion:**

Radiomic-based machine-learning has the potential to be utilized in differentiating atypical LGA from AA with reliable diagnostic performance.

## Introduction

Astrocytoma is one of the most common intracranial tumors characterized by rapid evolvement emphasizing the challenge for early diagnosis and intervention (1). Based on the 2016 World Health Organization (WHO) Classification of Tumors of the Central Nervous System, astrocytoma could be classified into low-grade astrocytoma (LGA, WHO II) and anaplastic astrocytoma (AA, also known as high-grade astrocytoma, WHO III) (2). The treatment and prognosis of these two subtypes are dramatically different. LGA is recognized as the subtype with good prognosis for most patients, while AA is the one with poor prognosis that the 5-year overall survival only reaches 28% when receiving conventional treatments (3–6).

The serum marker for the grading of astrocytoma has not been identified yet. As for radiological examination, brain magnetic resonance imaging (MRI) has been highly recommended with the ability to sensitively detect small lesions and accurately localize lesions. A common differentiator on the contrast-enhanced T1 weight imaging (T1C) is the presence of enhancement of AA and the absence of enhancement of LGA. However, high-grade gliomas can sometimes mimic the appearance of low-grade gliomas, and enhancement can also be seen in some cases of low-grade gliomas which indicates aggressive behavior (7–10). The meta-analysis by Abrigo and colleagues included seven studies with 115 solid and non-enhancing glioma patients, and evaluated the diagnostic capability of cerebral blood volume (CBV), which was expressed as ratio of tumoral CBV to normal white matter CBV (rCBV), in differentiating low-grade gliomas and high-grade gliomas (11). Results of this meta-analysis indicated that 7% to 34% low-grade glioma cases may be misdiagnosed as high-grade gliomas, and around half of high-grade glioma cases may be misdiagnosed as low-grade gliomas by a rCBV threshold of 1.75 (11). The interpretation of MRI may be challenging and must take into account the timing of surgery, previous radiation, corticosteroid use, and chemotherapy (12). Additionally, three quarters of AA are the consequences of LGA transformation, which leads to the similarity on images and makes the presurgical distinctiveness of LGA from AA difficult in some cases (13). Thus, this highlights the urgent requirement of novel technology to make the interpretation of MRI more accurate and reliable.

Radiomics is the method which can provide non-visual information by extracting quantitative texture features with mathematical formulas. Moreover, with quantitative statistics extracted from images, machine learning algorithms could be introduced in assisting clinical practitioners in their work, such as diagnostic differentiation, clinical grading, and prognosis prediction (14–16). Thus, in the current study, we introduced the radiomics-based machine learning algorithms in distinguishing atypical LGA from AA. The discriminative models were established with a set of texture parameters extracted from conventional MR images, and their diagnostic performances were evaluated for direct comparison.

## Methods

### Patient Selection

**Figure 1:**
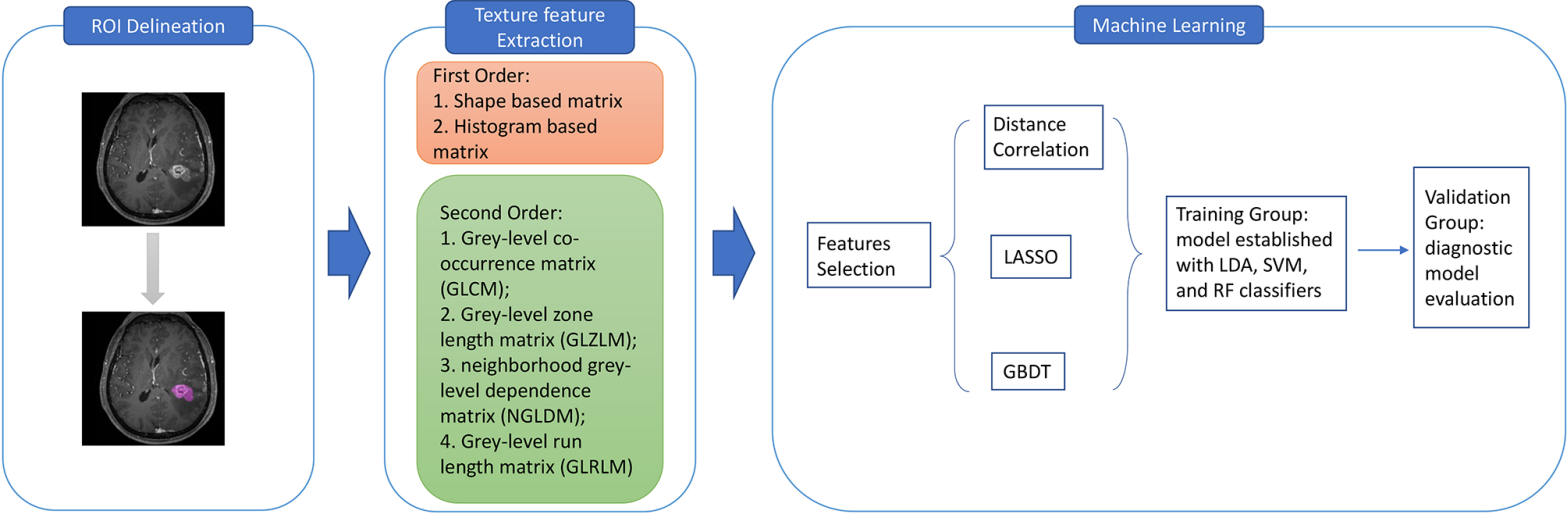
The workflow for texture features extraction and machine learning classification. ROI, region of interest; GLCM, grey-level co-occurrence matrix; GLZLM, grey-level zone length matrix; NGLDM, neighborhood grey-level dependence matrix; GLRLM, grey-level run length matrix; LASSO, least absolute shrinkage and selection operator; GBDT, gradient correlation decision tree; LDA, linear discriminant analysis; SVM, support vector machine; RF, random forest.

This study was performed in the Department of Neurosurgery and the Cancer Centre of West China Hospital. We initially reviewed the electronic medical records from April 2010 to December 2019 in our institution. The inclusion criteria of patients were as follows: 1) pathologically confirmation of LGA or AA by intraoperative frozen-section reports; 2) available high-quality pre-treatment MR images performed in the Department of Radiology. Exclusion criteria were: 1) recorded history of other types of intracranial diseases, such as head trauma, brain tumor, subarachnoid hemorrhage, cerebral apoplexy, and ischemic infarction; 2) incomplete electronic medical records; 3) patients had underwent treatment, such as surgery, radiotherapy and chemotherapy, prior to available MR images. The clinical parameters were also recorded, including age, gender, and pathology reports; and the presurgical MR images were exported from Picture Archiving and Communication Systems (PACS). The workflow of the current research is shown in **Figure 1**.

### MR Image Acquisition

Radiomics parameters should be extracted from the optimal MR sequence, defined as routine radiology examination which can provide precise segmentation of tumor tissue boundary. Therefore, T1C on the conventional MR sequence was selected. Other sequences like T1 weighted image (T1WI), T2 weighted image (T2WI), and fluid-attenuated inversion recovery (FLAIR) image were abandoned due to vague segmentation.

**Figure 2:**
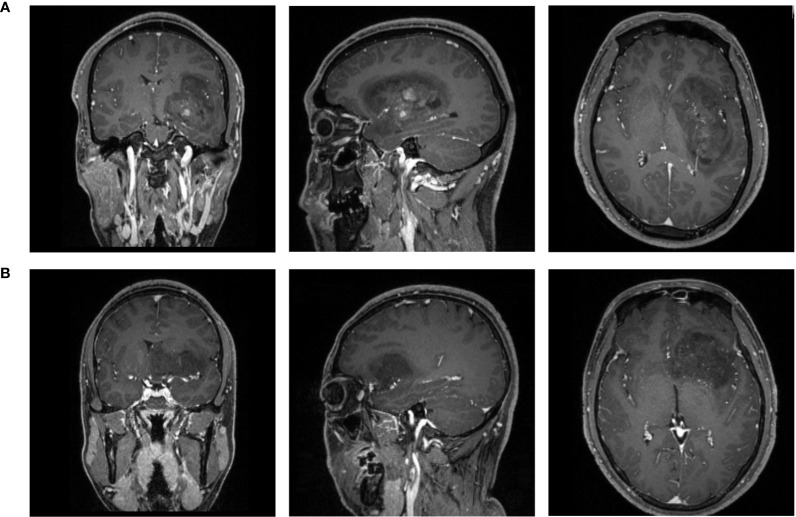
The examples of contrast-enhanced T1 magnetic resonance images of patients with **(A)** LGA; **(B)** AA. LGA, low grade astrocytoma; AA, anaplastic astrocytoma.

All patients underwent pre-treatment MR scan in the Department of Radiology in our institution with the 3.0T Siemens Trio Scanners before surgery. MPRAGE sequence was chosen to obtain high-resolution three-dimensional T1- weighted images. The parameters of scanners were: TR/TE/TI = 1900/2.26/900 ms, slice thickness = 1 mm, axial FOV = 25.6 × 25.6 cm^2^, Flip angle = 9°, and data matrix = 256 × 256. Gadopentetate dimeglumine (0.1 mmol/kg) was used as the contrast agent for contrast-enhanced images. The multi-directional data of T1C were collected during the continuous interval time of 90 to 250 s. **Figure 2** shows two examples of T1C sequence.

### Radiomics Analysis of MR Images

Two neurosurgeons participated in the texture features extraction using LifeX package (http://www.lifexsoft.org) and following the instructions on the website (17). The regions of interest (ROI) along the boundary of lesions in each layer were drown by these two neurosurgeons to obtain the 3D-based texture features. Necrosis and vessels within tissue were also included in ROI. The separation between adjacent structure invasion and peritumoral edema band was carefully identified from the primary tumor with the different pattern in contrast enhancement. The texture features were calculated automatically from two orders with default software protocol settings. In the first order, the calculation was based on shape- and histogram-based matrices; and in the second order, the calculation was based on grey-level co-occurrence matrix (GLCM), grey-level zone length matrix (GLZLM), neighborhood grey-level dependence matrix (NGLDM), and grey-level run length matrix (GLRLM), which plays a major role in the quantification of radiomic features. Finally, a total number of 40 features were extracted for further deployment in machine learning algorithms.

### Human Reader Assessment

To evaluated the diagnostic performance of human readers with naked eyes, two researchers independently made diagnosis of AA or LGA based on the contrast-enhancement pattern on T1C images and their experience. The human readers were blinded to the patient characteristics and the pathological reports. The diagnostic accuracy of LGA and AA and the overall diagnostic accuracy were calculated for further analysis.

### Classification Model Establishment

The selection of proper features for algorithms was necessary due to the reason that excessive features could lead to inevitable overfitting. Moreover, the selected features could directly affect the diagnostic performance of the algorithms. Thus, we adopted three selection methods, including Distance Correlation, least absolute shrinkage, and selection operator (LASSO), and Gradient Boosting Decision Tree (GBDT). In the current study, the classification models were established based on three algorithms, including Linear Discriminant Analysis (LDA, also known as Fisher Linear Discriminant), Support Vector Machine (SVM), and Random Forest (RF). Then nine models were established with varied combinations of three selection methods and three classification algorithms.

For the deployment of classification algorithms, patients were divided into the training group and the validation group at a ratio of 4:1. For each model, sensitivity, specificity, accuracy, and area under the receiver operating characteristic curve (AUC) of the training group and the validation group were calculated to evaluate their discriminative performance. This progress was repeated 100 times to obtain the realistic distribution of accuracies. The feature selection and classification procedures were applied using Scikit-learn 0.22, a Python module for machine leaning, with parameters suggested by the developers.

## Results

### Patient Selection

A total number of 175 patients were enrolled in the current study according to the inclusion and exclusion criteria. For patients diagnosed before 2016, we made corrections on their diagnosis based on their pathology reports according to the new WHO criteria. Ninety-five patients were diagnosed with LGA, and 80 patients were diagnosed with AA. Baseline characteristics of patients and diagnostic accuracy of human readers are shown in **Table 1**. For LGA patients, the median age was 32 years (range, 1–64 years), and the male ratio was 42/95 (44.2%). And for AA patients, the median age was 39 years (range, 6–69 years), and the male ratio was 43/80 (53.8%). The mean time between the MR scan and surgery was 6.7 and 7.2 days for LGA and AA patients, respectively. The mean diagnostic accuracy of the two human readers for LGA was 75.2%, and that for AA was 62.5%. The mean overall accuracy of human readers was 69.5%.

**Table 1:** Baseline characteristics of patients and diagnostic accuracy of human readers.

Characteristics	LGA (n=95)	AA (n=80)	All (n=175)
Age (y, range)	32 (1–64)	39 (6–69)	35 (1–69)
Sex (%)			
Male	42 (44.2)	43 (53.8)	85 (48.6)
Female	53 (55.8)	37 (46.2)	90 (51.4)
Time between MR scan and surgery (d)	6.7	7.2	6.9
Diagnostic accuracy (%)			
Human reader 1	75.8	60.0	68.6
Human reader 2	74.7	65.0	70.3

LGA, low grade astrocytoma; AA, anaplastic astrocytoma.

### Discriminative Ability of Models

**Figure 3:**
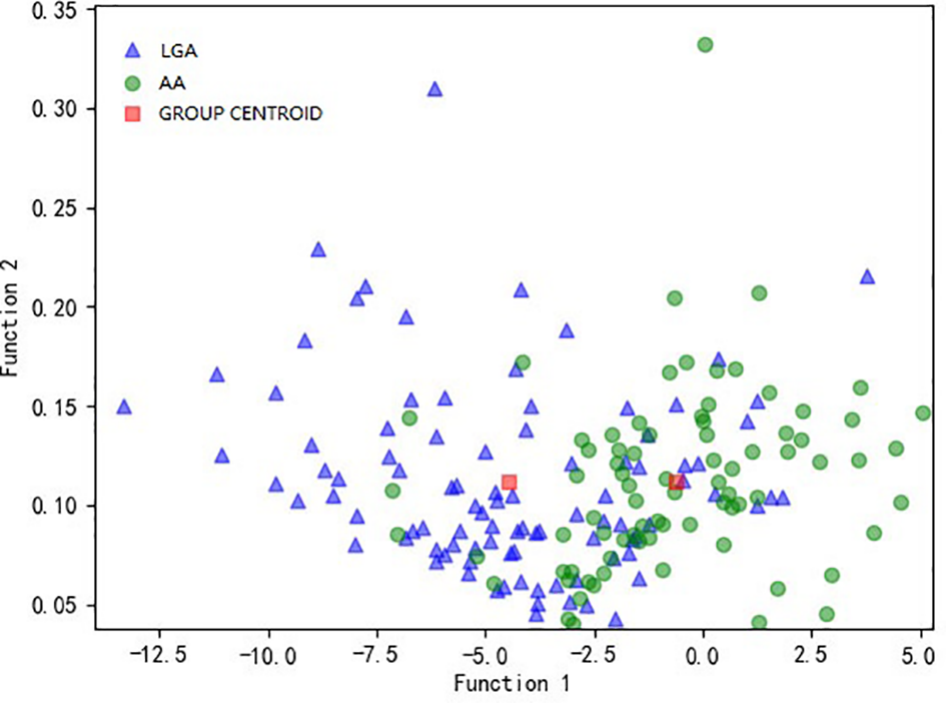
Relationship between the canonical discriminant functions of LASSO + LDA model for LGA and AA groups and the group centroids. LASSO, least absolute shrinkage and selection operator; LDA, linear discriminant analysis; LGA, low grade astrocytoma; AA, anaplastic astrocytoma.

**Figure 4:**
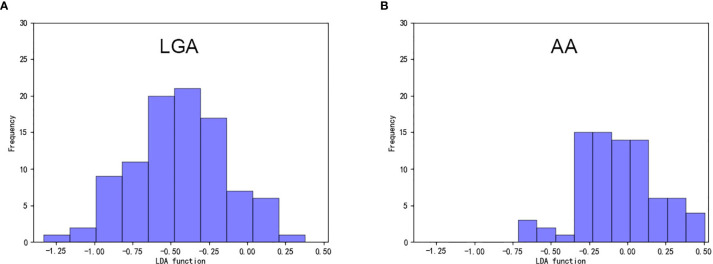
Example of the distribution of the direct LDA canonical function determined for **(A)** LGA and **(B)** AA in one cycle. LDA, linear discriminant analysis; LGA, low grade astrocytoma; AA, anaplastic astrocytoma.

Among the nine models we evaluated, LASSO + LDA was chosen as the optimal one with the highest AUC in the validation group. In the training group, the sensitivity, specificity, accuracy, and AUC of LASSO + LDA were 0.782, 0.717, 0.752, and 0.835, respectively; and in the validation group, the sensitivity, specificity, accuracy, and AUC were 0.751, 0.703, 0.729, and 0.825, respectively (**Table 2**). Results showed that the diagnostic performance of LASSO + LDA was superior to human readers. The canonical discriminant functions of LASSO + LDA model for LGA, AA groups, and the group centroids are presented in **Figure 3**. **Figure 4** is one example of the 100 validation cycles of LASSO + LDA model, which shows the distribution of the direct LDA canonical function determined for LGA and AA.

**Table 2:** Results of the discriminative models in distinguishing LGA (low grade astrocytoma) From AA (anaplastic astrocytoma) in the the training and the validation group.

Model	Training Group				Validation Group			
	Sensitivity	Specificity	Accuracy	AUC	Sensitivity	Specificity	Accuracy	AUC
Distance Correlation + LDA	0.745	0.705	0.727	0.801	0.746	0.654	0.700	0.799
Distance Correlation + SVM	0.659	0.765	0.707	0.771	0.721	0.821	0.766	0.808
Distance Correlation + RF	0.761	0.717	0.741	0.825	0.770	0.720	0.740	0.821
LASSO + LDA	0.782	0.717	0.752	0.835	0.751	0.703	0.729	0.825
LASSO + SVM	0.713	0.626	0.673	0.743	0.756	0.640	0.706	0.761
LASSO + RF	0.756	0.788	0.771	0.859	0.719	0.783	0.746	0.817
GBDT + LDA	0.767	0.749	0.759	0.833	0.707	0.730	0.714	0.773
GBDT + SVM	1	1	1	1	Error	0.537	0.537	0.5
GBDT + RF	0.754	0.769	0.761	0.855	0.751	0.789	0.766	0.815

AUC, area under curve; LDA, linear discriminant analysis; SVM, support vector machine; RF, random forest; LASSO, least absolute shrinkage and selection operator; GBDT, gradient boosting decision tree.

Detailed performance in the training group and validation group of all the nine models is shown in **Table 2**. Actually, the results suggested that all of the three algorithm-base models represented similar feasible performance when combined with a suitable selection method except for the GBDT + SVM model. The optimal SVM-based and RF-based models represented similar diagnostic performance with LDA-based models that the highest AUC of SVM-based models in the validation group was 0.808 (**Table 2**), and that of RF-based models was 0.821 (**Table 2**). However, overfitting was observed in the classification algorithm of SVM when it was combined with GBDT.

## Discussion

In the current study, we investigated the ability of pattern recognition techniques with radiomics features extracted from conventional MRI in discriminating atypical LGA from AA. Nine models were evaluated based on three classification algorithms and three selection methods. The results suggested that the machine learning technology could be potentially utilized in presurgical astrocytoma grading with promising ability.

Astrocytoma is one of the traditional basic subtypes of diffuse grade II and III gliomas. The accurate diagnosis is clinically important because the surgical planning and therapeutic strategy are significantly different between LGA and AA. The descriptions of their characteristics on MR images are as follows: the classic LGA tumor tissues do not show enhancement after contrast administration; while AA tissues are also not usually contrast enhanced, and if they are, they represent as a focal, nodular, or patchy appearance (18). However, the enhancement pattern can also be seen on images of atypical LGA as a suggestive signal of malignant transformation and high growth rate of the tumor diameter (19). This special transforming status makes the astrocytoma grading disturbing and vulnerable when interpreting the conventional MRI, even for experienced radiologists (20).

Radiomics analysis can provide non-visual information by statistically calculating the voxels of images reflecting the tumor pathology process and abnormal microenvironment (21). It is able to transform the images into analyzable statistics with mathematical calculation, which could be further applied in machine learning technology. This set of methods has been widely explored in clinical diagnosis, tumor grading, treatment prediction, and survival prediction by previous researchers (16, 22–27). For example, the study applying RF classifier in the discrimination between primary central nervous system lymphoma and atypical glioblastoma represented satisfactory diagnostic ability with AUC of 0.921 (27). Another study applied SVM-based algorithm in glioma grading, concluding that SVM classifier feasibly achieves multiclass glioma grading as well as low classification error for intermediate (16).

However, the pervasive limitation for these studies was that the establishment of the models was seemingly arbitrary. Most studies focused on only one type of algorithms, and the adoption of feature selection method was not persuasive enough due to the lack of direct comparison. The current study established a set of machine learning-based models to discriminate LGA and AA with a relatively large number of radiomics features extracted from T1C images. The three algorithms we used in discrimination were LDA, SVM, and RF. These classification algorithms represented different types of classifiers. LDA is the classic linear classifier, which separates two or more classes by a linear combination of features (28). SVM is the classic non-linear classifier, which constructs a decision hyperplane and separates classes by maximizing the distance between the training samples of classes and the hyperplane (28). And RF is the ensemble learning classifier, which is realized by performing a weighted integration of the predictive probabilities of de-correlated trees (29, 30). Moreover, both LDA and SVM represent the state-of-the-art in pattern recognition classifier applications (31). The choice of suitable algorithms is complicated. In general, as performance improves, the complexity and computational time would increase at the same time. Therefore, the researchers should choose the classification algorithm based on a trade-off between performance and computational burden, especially in embedded systems (28). Based on this point, we chose three classification algorithms to make an evaluation. The results represented that the performance of all three classifiers could have similar, promising diagnostic ability when combined with suitable selection method.

As mentioned above, the selection method also played an important role in the performance of classifiers. The relatively large number of parameters made it more likely to find the optimal parameters but also increased the difficulty in feature selection. Previous studies performed feature selection with varied methods, including Student’s t test with recursive feature elimination, Mann-Whitney U test with AUC of ROC, entropy-based discretization, or random forest (27, 32–34). We adopted three feature-selection methods (Distance Correlation, LASSO, and GBDT) in the current study.

The results of this study suggested that LASSO + LDA was the optimal discriminative model with the best performance. LASSO was recommended due to the ability of producing interpretable models when exhibiting the stability of ridge regression simultaneously. It was regarded as the nonlinear variable selection method for neural network with the advantage of minimizing the common sum of squared errors and the superior performance over other state-of-the-art variable selection methods (35). This might provide a potential underlying mechanism for our results. The LDA and RF classification algorithms both presented with consistent diagnostic performance when combining with different feature selection methods. While overfitting was observed in the model of GBDT + SVM, we suspect that the overfitting of this model was caused by the over-dependence of SVM on its kernel functions and support vectors. However, our results must be interpreted cautiously that the additional information from comparison of machine learning techniques was limited given that variance in AUC maybe partially attributed to the statistical group and all classifier/feature selection methods investigated seem perform quite comparably. Our study should be regarded as the hypothesis generation for future studies with large study population.

There were some limitations in our study. First, the study was a single-institution, retrospective study with limited patients enrolled. A research with a larger study population would be required to validate our results in the future. Second, texture features extraction was only performed on T1C images, while other conventional sequences (like T1WI, T2WI, and FLAIR) and advanced MR techniques were not investigated. Third, there was no independent validation group in our study selected from other institution. This point was not performed because that texture features could be unpredictable when extracted from images acquired with various scanners and/or protocols. Considering the analysis protocol and image processing procedure were open-source packages, the results should be validated and reproduced in a future study.

## Conclusion

Evidence of this study indicated that radiomics-based machine learning has the potential to be utilized in the preoperative differential diagnosis between atypical LGA and AA with reliable diagnostic performance. We established high-performance prediction models based on selection methods and classification algorithms, indicating that this non-invasive approach has the potential to assist image diagnosis and aid clinical decision-making.

## Data Availability Statement

The data sets generated for this study are available on request to the corresponding authors.

## Ethics Statement

Written informed consent was obtained from the individual(s), and minor(s)’ legal guardian/next of kin, for the publication of any potentially identifiable images or data included in this article.

## Author Contributions

BC, CC, and YT designed the study. BC and CC included eligible patients, obtained medical records and MR images of each patient and performed texture analysis. JW established the models and did other statistical analysis. BC, CC, XM, and JX made substantial revisions to the manuscript. All authors contributed to the article and approved the submitted version.

## Funding

This study was supported by 1.3.5 Project for Disciplines of Excellence, West China Hospital, Sichuan University (ZYJC18007).

## Conflict of Interest

The authors declare that the research was conducted in the absence of any commercial or financial relationships that could be construed as a potential conflict of interest.
